# A novel role of h2‐calponin in regulating whole blood thrombosis and platelet adhesion during physiologic flow

**DOI:** 10.14814/phy2.12228

**Published:** 2014-12-03

**Authors:** Patrick C. Hines, Xiufeng Gao, Jennell C. White, Ashley D'Agostino, Jian‐Ping Jin

**Affiliations:** 1 Division of Pediatric Critical Care Medicine, Department of Pediatrics, Wayne State University School of Medicine, Detroit, Michigan; 2 Department of Physiology, Wayne State University School of Medicine, Detroit, Michigan; 3 Children Hospital of Michigan, Detroit Medical Center, Detroit, Michigan

**Keywords:** Calponin, microfluidics, platelets, shear, thrombosis

## Abstract

Calponin is an actin filament‐associated protein reported in platelets, although the specific isoform expressed and functional role were not identified. The h2‐calponin isoform is expressed in myeloid‐derived peripheral blood monocytes, where it regulates adhesion. Our objective was to characterize the presence and function of the h2 isoform of calponin in platelets. H2‐calponin was detected in human and mouse platelets via Western blotting. Immunofluorescent staining demonstrated h2‐calponin and actin colocalized in both human and wild‐type mouse platelets at rest and following collagen activation. The kinetics of platelet adhesion and whole blood thrombosis during physiologic flow was evaluated in a microfluidic flow‐based thrombosis assay. The time to initiation of rapid platelet/thrombus accumulation (lag time) was significantly longer in h2‐calponin knockout versus wild‐type mouse blood (130.02 ± 3.74 sec and 72.95 ± 16.23 sec, respectively, *P* < 0.05). There was no significant difference in the rate of platelet/thrombus accumulation during the rapid phase or the maximum platelet/thrombus accumulation. H2‐calponin knockout mice also had prolonged bleeding time and blood loss. H2‐calponin in platelets facilitates early interactions between platelets and collagen during physiologic flow, but does not significantly affect the rate or magnitude of platelet/thrombus accumulation. H2‐calponin knockout mice take 2.3 times longer to achieve hemostasis compared to wild‐type controls in a tail bleeding model. The ability to delay platelet accumulation without inhibiting downstream thrombotic potential would be of significant therapeutic value, thus h2‐calponin may be a novel target for therapeutic platelet inhibition.

## Abstract (precis)

This study provides the first report that the h2 isoform of calponin is present in platelets, facilitates early interactions between platelets and collagen during physiologic flow, but does not significantly affect the rate or magnitude of platelet/thrombus accumulation. h2‐calponin knockout mice take 2.3 times longer to achieve hemostasis compared to wild‐type controls in a tail bleeding model. The ability to delay platelet accumulation without inhibiting downstream thrombotic potential would be of significant therapeutic value, thus h2‐calponin may be a novel target for therapeutic platelet inhibition.

## Introduction

Platelet function is a critical determinant of thrombosis and hemostasis, thus it is critical to understand the molecular factors that regulate platelet activity. The function of actin cytoskeleton is a major determinant of platelet activity because actin closely associates with the cytoplasmic regions of important adhesion receptors on the surface of platelets (Baig et al. 2009; Gao et al. 2009; Cerecedo et al. 2010; Gonzalez et al. 2012; Pertuy et al. 2014). Integrin *α*2*β*1 is an adhesion receptor on the platelet surface that binds to collagen, and thus facilitates platelet adhesion and initiation of a hemostatic plug (Kehrel et al. 1998; Clemetson et al. 1999; Nieswandt et al. 2001; Yan et al. 2001; Miura et al. 2002; Petrich et al. 2007a,b; Smethurst et al. 2007). The ability of platelets to effectively bind to collagen during physiologic flow conditions is in part dependent on the association of actin with the cytoplasmic tail of the *β*1 subunit through binding partners such as talin (Banno et al. 1995; Mitsios et al. 2010). The discovery of other endogenous regulators of the actin cytoskeleton may reveal attractive therapeutic targets.

Calponin is an actin filament‐associated protein expressed in both smooth muscle and nonmuscle cells and exists in three distinct isoforms, h1 (basic), h2 (neutral), and h3 (acidic). Calponin was initially observed in bovine platelets by Takeuchi et al. (1991), and shown to colocalize with actin during resting and stimulated conditions. Calponin was later identified in human platelets by Meyer et al. (1996), where colocalization with actin selectively occurred in activated platelets and this colocalization was independent of the phosphorylation state. Nevertheless, isoform specificity of platelet calponin has never been described in humans or other species. Although these previous studies suggest that calponin may regulate contraction and secretion of platelets in the absence of phosphorylation, a functional role for calponin in platelet thrombosis has not been clearly demonstrated.

Compared to the more restricted tissue distribution of h1 (basic) and h3 (acidic) calponin isoforms, h2 (neutral) calponin is expressed broadly in various tissue types, including developing and remodeling smooth muscles, epidermal keratinocytes, fibroblasts, lung alveolar cells, endothelial cells, and white blood cells of myeloid lineage (Wu and Jin 2008). H2‐calponin has a clear functional role in monocytes, which like platelets are peripheral blood cells of the myeloid lineage (Huang et al. 2008). Previous studies in h2‐calponin knockout (KO) mice have demonstrated that h2‐calponin can regulate macrophage motility and phagocytosis. Additionally, h2‐calponin KO macrophages had a higher rate of proliferation and faster migration, and had reduced spreading in adhesion culture together with decreased level of tropomyosin in the actin cytoskeleton when compared to h2‐calponin‐positive macrophages. The lack of h2‐calponin also resulted in significantly increased phagocytic activity, suggesting a novel mechanism in the regulation of macrophage function (Huang et al. 2008).

Like monocytes and macrophages, platelets are also of the myeloid lineage and have very well‐described actin‐mediated functions, such as spreading on adhesive surfaces, adhesion receptor localization, and clot retraction (Santos‐Martínez et al. 2011). These anucleated, transcriptionally inactive megakaryocyte fragments have a very elaborate posttranslational system for regulating actin function (Neeves et al. 2008; Gao et al. 2009; Stalker et al. 2013). As a result, we hypothesized that h2‐calponin may play a role in regulating cytoskeletal association with platelet adhesion receptors. In this report, we describe the presence, localization, and functional role of h2‐calponin in platelets.

## Materials and Methods

All animal procedures were approved by the Institutional Animal Care and Use Committees of Wayne State University and were conducted in accordance with the Guiding Principles in the Care and Use of Animals. The procedure to develop calponin KO mice has been previously described (Huang et al. 2008). Human blood was obtained from discarded and deidentified freshly drawn sodium citrate anticoagulated venous blood from the Children's Hospital of Michigan infusion center in accordance of the Wayne State University Institutional Review Board.

### Isolation of platelets, leukocytes, and erythrocytes from whole blood

Isolation of platelets, leukocytes, and erythrocytes from human and mouse whole blood was performed in a similar manner as previously described (Maxwell et al. 2004), with exceptions noted for mouse blood. Briefly, citrated whole blood was layered onto an equal volume of Histopaque 1077 (Sigma Aldrich, St. Louis, MO) or Hitopaque 1083 (Sigma Aldrich) for human or mouse blood, respectively. Layered whole blood was then centrifuged at 400 × *g* for 30 min (20 min for mouse blood) at 25°C. Platelet‐rich plasma (PRP) and the leukocyte‐containing buffy coat were removed and placed into centrifuge tubes. The erythrocyte pellet was washed three times at 250 × *g* for 10 min. The PRP was centrifuged at 250 × *g* for 10 min and centrifuged again at 800 × *g* (1300 × *g* for mouse PRP) for 10 min to isolate the platelets. The leukocyte‐containing buffy coat was centrifuged at 250 × for 10 min to isolate leukocytes.

### SDS‐polyacrylamide gel electrophoresis (PAGE) and Western blot analysis

SDS‐PAGE and Western blotting analysis were utilized to examine the expression of calponin in human and mouse leukocytes, erythrocytes, and platelets. Briefly, total protein of cell pellets was extracted by sonication in SDS gel electrophoresis sample buffer containing 2% SDS and 150 mmol/L dithiothreitol (DTT). The protein extract was then heated at 80°C for 5 min, centrifuged at 14,000 rpm in a microcentrifuge (Beckman Coulter Microfuge 18, Danvers, MA) for 5 min to precipitate insoluble residues. Samples were resolved on 12% Laemmli gel with an acrylamide:bisacrylamide ratio of 29:1. Sample integrity and protein contents were confirmed by visualization on gels fixed and stained with Coomassie Blue R250.

Unfixed duplicate gels were electrically blotted on nitrocellulose membrane using a Bio‐Rad semi‐dry transfer apparatus for Western blotting analysis. After blocking with 1% bovine serum albumin in Tris‐buffered saline for 30 min, the membrane was incubated with a rabbit anti‐calponin antiserum, RAH2, at 1:2000 dilutions in Tris‐buffered saline containing 0.1% bovine serum albumin to examine the expression of calponin. The blots were washed with Tris‐buffered saline containing 0.05% Tween‐20 and incubated with alkaline phosphatase‐labeled goat anti‐rabbit IgG at a 1:5000 dilution (Santa Cruz Biotechnology, Santa Cruz, CA). After the final washes, the blots were processed for 5‐bromo‐4‐chloro‐3‐indolyl phosphate and nitro blue tetrazolium chromogenic substrate reaction as previously described (Huang et al. 2008).

### Immunofluorescence analysis

For immunofluorescence analysis, cells were cytospun onto slides using a Shandon cytospin III cytocentrifuge and then fixed with 4% paraformaldehyde in phosphate‐buffered saline (PBS) at room temperature for 10 min and perforated with 1% Triton X‐100 in PBS for 4 min. After treatment with 1% bovine serum albumin in PBS for 30 min, the cells were incubated at 4°C overnight with a rabbit anti‐h2‐calponin antibody RAH2 or normal rabbit serum (NS) at 1:200 dilution. A second antibody, FITC‐conjugated anti‐rabbit IgG (Sigma) was utilized at a 1:200 dilution and incubated at room temperature for 60 min in the dark. Filamentous actin was stained with phalloidin–tetramethylrhodamine B isothiocyanate (phalloidin‐TRITC, Sigma) at 1:100 dilution. Coverslips were mounted onto slides with ProLong antifade with DAPI (for nuclear staining) mounting medium (Invitrogen, Carmillo, CA) and air‐dried in the dark for 24 h, sealed with varnish. Cells were examined with a Zeiss Axioimager Z1 fluorescence microscope (Carl Zeiss, Oberkochen, Germany) and a 63× , numerical aperture 1.4 oil immersion objective lens. Images were acquired using a Zeiss AxioCam MRm monochrome cooled‐CCD camera and analyzed using Axiovision version 4.8 software (Carl Zeiss).

### Mouse whole blood platelet adhesion and aggregation assay

The Bioflux 1000Z well‐plate microfluidic (WPM) flow adhesion system (Fluxion Biosciences, San Francisco, CA) used for flow adhesion/thrombosis assays has been previously described for assessment of platelet adhesion/thrombosis (Conant et al. 2011). Individual channels of WPM (48‐well) (Fluxion Biosciences) were coated with rat‐tail collagen type I (Sigma) at 200 *μ*g/mL in 0.02 mol/L acetic acid by perfusion at 2 dynes/cm^2^ for 5 min. The 48‐well plates were incubated at room temperature for 1 h. Coated channels were perfused with Dulbecco's phosphate‐buffered saline with calcium and magnesium (DPBS) at 5 dynes/cm^2^ for 10 min to wash off unbound substrate. Bovine serum albumin (0.5%) in DPBS was used to block the coating by perfusion at 5 dynes/cm^2^ for 10 min.

Blood was collected from the inferior vena cava of h2‐calponin KO and wild‐type mice anesthetized with 50 mg/kg of sodium pentobarbital and anticoagulated with 0.109 mol/L (3.2% W/V) sodium citrate with a ratio of blood to anticoagulant volume of 6:1. Blood was labeled with 2 *μ*mol/L 3,3′‐dihexyloxacarbocyanine iodide (DiOC6 (Cerecedo et al. 2010)) at room temperature for 30 min. The labeled blood was diluted with equal volume of Hank's balanced salt solution (HBSS) containing 1 mmol/L of calcium chloride and 0.5 mmol/L of magnesium chloride (Sigma) prior to the thrombosis assay. The diluted sample was run through the channels at a shear rate of 15 dynes/cm^2^ and a pulse frequency of 1.67 Hz.

Platelet adhesion and thrombus accumulation was visualized within the viewing area of the microfluidic channels with a fluorescence microscope (Axioobserver Z1, Carl Zeiss) at 100× magnification. Serial fluorescent images were acquired with a high‐resolution CCD camera (QImaging, Surrey, BC, Canada) from three positions, in the center of each channel, within the viewing window at image acquisition settings (interval, exposure time and gain). The fluorescent images of adherent platelets and thrombi were analyzed for both total fluorescent intensity (TFI) and total area covered (TAC) using the Bioflux Montage image analysis software. Total fluorescent intensity (TFI) was quantitated at each time point and the change in TFI of the entire image represents both dynamic platelet adhesion and dynamic thrombus accumulation. The kinetics of individual thrombi formation was also analyzed. To control for changes in activation state of platelets that occur as they flow along the horizontal axis between input and output ends of microfluidic channel, only images acquired midway between the input and output wells were analyzed (Neeves et al. 2008). To control for regional differences in shear induced by drag along the edges of the microfluidic channels, only thrombi located within the vertical middle third of the microfluidic channel were selected (Fig. 3B) (*n* = 33 individual thrombi from four separate WT and 33 individual thrombi from four separate KO mice). In the context of individual thrombi analysis, TFI was used to assess growth in the z‐plane, and TAC was used to assess thrombus growth in the x,y‐plane.

### Image analysis

The fluorescence images of adherent platelets and thrombi were analyzed using the Montage image analysis software. Total fluorescent intensity and area covered by thrombi were quantitated at each time point and the change in fluorescent intensity over time was used to calculate the kinetics of platelet adhesion and aggregation. A best‐fit curve for the kinetics was used to calculate the lag time, defined as the initiation of rapid platelet adhesion / thrombosis. Briefly, quadratic model was found as best‐fit for observed data sets. Then quadratic formula for each set of observed data (per channel) was calculated with SPSS Statistical packet (SPSS/21.0; IBM, Armonk, NY). Lag time was determined by solving At^2^ + Bt + C = 0, where the *t* in the ascending branch of the curve was defined as lag time.

### Mouse tail bleeding assay

Three pairs of sex‐ and age‐matched wild type and h2‐calponin knockout mice were anesthetized with sodium pentobarbital at 50 mg/kg body weight. The distal 10‐mm segment of tail was amputated with a surgical blade and the tail was submerged in 50 mL of isotonic saline prewarmed to 37°C. The time to bleeding cessation was recorded up to 20 min. During start and stop bleeding cycles, the total sum of bleeding times was used up to 20 min [2]. Body weight was measured before and after the bleeding test (the amputated tail tip was also weighed with the mouse after the bleeding test for weight accuracy). The blood‐saline mixture was mixed well before being photographed. Cropped images from each corresponding tube were obtained using Photoshop CS3 software. The volume of blood loss during the test period was estimated from reduction in body weight.

### Data analysis

The SPSS statistical packet (SPSS/21.0, IBM) was used for curve‐fitting and data analysis. Lag time was analyzed using Student's *t*‐test for comparison of two unpaired means. Total intensity and slope were analyzed using two‐way repeated measure ANOVA for comparison of unpaired means with time. Threshold for significance was set as *P* < 0.05. Values are presented as means ± SE.

## Results

### Identification and localization of h2‐calponin in human platelets

**Figure 1.**
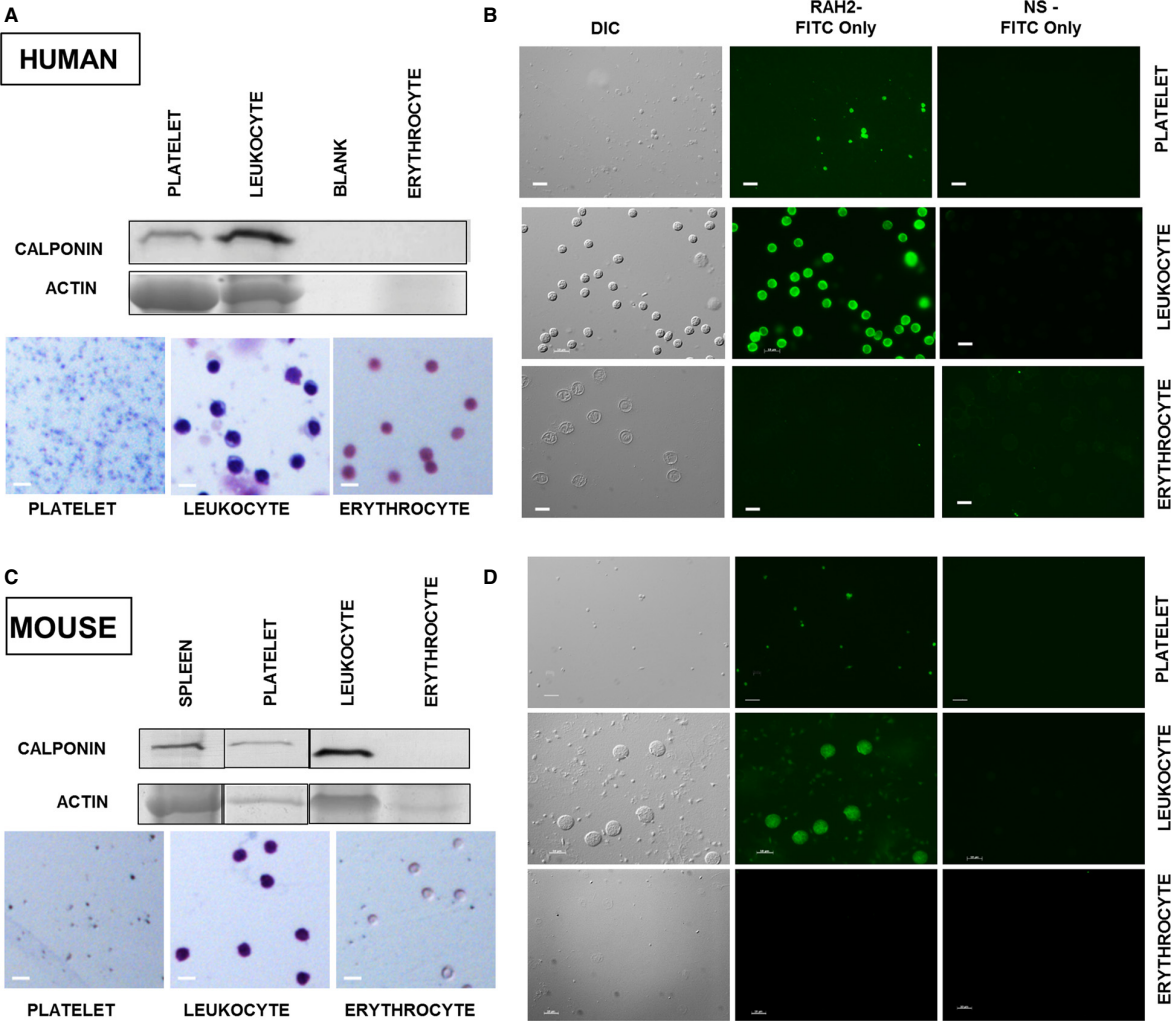
Identification of h2‐calponin in human (A, B) and mouse platelets (C, D). (A, C): Platelets, leukocytes, and erythrocytes were prepared on slides to assess for contamination. Lysates from platelet preparations were resolved on SDS‐PAGE along with erythrocyte and leukocyte lysates (internal negative and positive controls, respectively). H2‐calponin was probed (RAH2 primary antibody and, alkaline phosphate‐labeled anti‐rabbit IgG second antibody (B, D): Platelets, leukocytes, and erythrocytes were examined by immunofluorescence. H2‐calponin was visualized using RAH2 antibody followed by FITC‐conjugated second antibody (central column). Normal rabbit serum (NS) was used as primary antibody control (right column). Bright‐field images (left column) were obtained using a differential interference contrast (DIC) filter. Size bars indicate 10 μm.

H2‐calponin is an important regulator of actin cytoskeleton in multiple nonmuscle cell types, including epithelial cells (Hossain et al. 2005, 2006), endothelial cells (Tang et al. 2006), fibroblasts (Hossain et al. 2005), and myeloid phagocytes (Huang et al. 2008). Although calponin has been identified in bovine and human platelets (Takeuchi et al. 1991; Meyer et al. 1996), the specific isoform has not been described. We identified h2‐calponin in isolated human platelets by Western blotting (Fig. 1A) and immunofluorescence (Fig. 1B). H2‐calponin was not detected in human erythrocytes (Fig. 1A). As an internal positive control, the presence of h2‐calponin was also confirmed in a leukocyte‐enriched preparation (Fig. 1A). Representative photomicrographs confirmed that there was negligible contamination in the platelet, leukocyte, and erythrocyte preparations (Fig. 1A). Immunofluorescence staining identified h2‐calponin in resting human platelets and leukocytes, whereas erythrocytes from identical patients did not stain for h2‐calponin (Fig. 1B).

**Figure 2.**
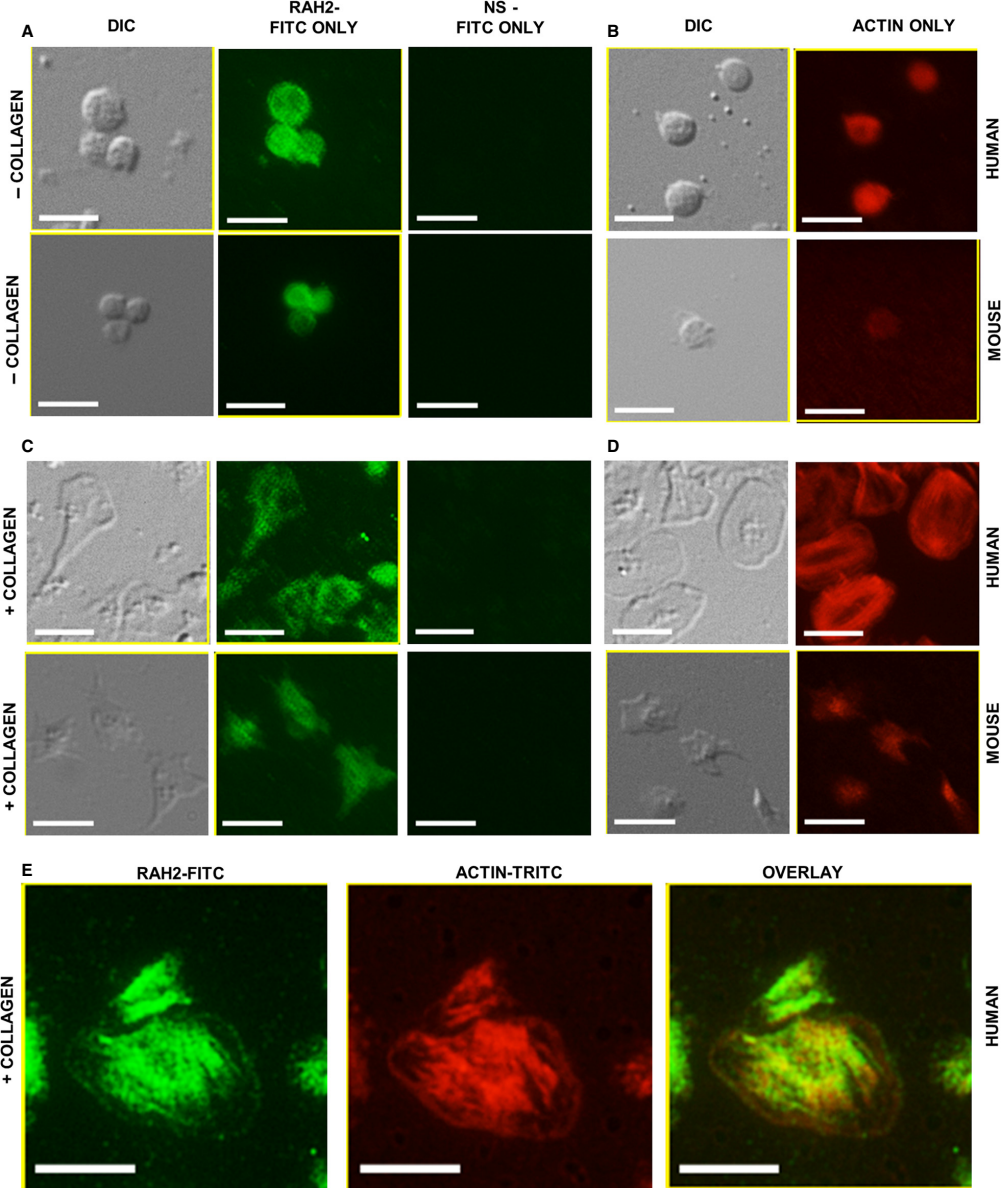
Distribution of h2‐calponin in human and mouse resting versus activated platelets. Resting platelets showing (A) RAH2 immunofluorescence and (B) actin staining. Platelets activated on a collagen substrate showing (C) RAH2 immunofluorescence and (D) actin staining. (E) Colocalization of h2‐calponin and actin in activated human platelets. Green = h2‐calponin, Red = actin, yellow = colocalization. Images were cropped from their corresponding originals and enlarged to display structures of interest. Size bar indicates 5 μm.

The distribution of h2‐calponin was analyzed in human platelets during both resting and activated conditions as previously described. H2‐calponin was primarily distributed throughout the cytoplasm in resting human platelets (Fig. 2A). F‐actin had a similar homogeneous distribution throughout resting human platelets (Fig. 2B). Activated human platelets displayed characteristic lamellipodia while spreading on immobilized collagen, and h2‐calponin had a focal punctate distribution within the leading edge, interspersed with actin. H2‐calponin and F‐actin more consistently colocalized throughout the centrally anchored region in human platelets (Fig. 2E).

### Identification and localization of h2‐calponin in mouse platelets

The availability of h2‐calponin KO mice provides a model to understand the function of h2‐calponin in platelets. We confirmed the presence of h2‐calponin in mouse platelets via Western blotting (Fig. 1C) and immunofluorescence (Fig. 1D). The representative photomicrographs of the isolated mouse platelets, leukocytes, and erythrocytes confirmed that there was no significant contamination in these cell preparations (Fig. 1C).

The distribution of h2‐calponin was analyzed in mouse platelets during both resting and collagen‐activated conditions. Resting platelets were fixed and permeabilized to minimize any opportunity for activation during slide preparation. Platelets intended for activation were incubated on collagen‐coated slides, followed by fixation and permeabilization as described in the materials and methods section. H2‐calponin was distributed throughout the cytoplasm in resting mouse platelets with no distinct membrane association (Fig. 2A). Activated mouse platelets displayed characteristic spreading on immobilized collagen, and h2‐calponin appeared homogenously distributed within the cytoplasmic projections and throughout the body of each platelet (Fig. 2A). F‐actin appeared to have a similar homogeneous distribution throughout both resting and collagen‐activated platelets (Fig. 2B).

### Assessment of platelet function in h2‐calponin KO mice versus wild‐type controls

**Figure 3.**
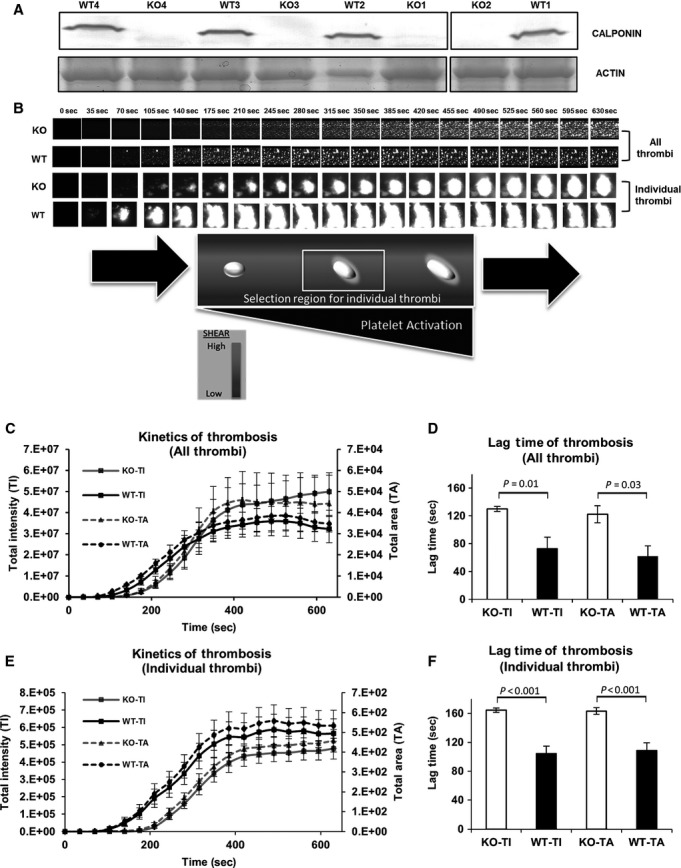
Whole blood samples from h2‐calponin KO mice demonstrate a prolonged lag time compared to WT in a microfluidic flow‐based thrombosis assay. (A) Genotypes of four matched mouse pairs (WT vs. h2‐calponin KO) were confirmed by presence or absence of h2‐calponin on Western blot (Gel/blot images were cropped/sliced from the same gel/blot). Whole blood preparations, labeled with 2 μmol/L DIOC6, from wild‐type or calponin KO mice were flowed over an immobilized collagen substrate at a shear stress of 15 dynes/cm², pulse frequency of 1.67 Hz, and a constant temperature of 37°C. (B) Photomicrographs from a representative microfluidic flow‐based thrombosis assay were taken at 35 sec intervals during a 10 min flow period. The top two rows show all interactions captured within the central viewing region over time. The bottom two rows show the growth of a representative single thrombus over time. Total (fluorescent) intensity (TI) and area covered by thrombi (TA) over time were graphed in (C) (overall thrombi) and in (E) (individual thrombi), representing the kinetics of platelet / thrombus accumulation. Each point on the graph represents the average intensity for WT (*n* = 4) and h2‐calponin KO group (*n* = 4). D. Graph of the lag time of overall platelet / thrombus accumulation. The lag time is significantly longer in the h2‐calponin KO group compared to the WT group based on total intensity (TI) and total area (TA) covered by thrombi. (F) The lag time of individual thrombus accumulation was significantly longer in the h2‐calponin KO compared to the WT group based on total intensity, and on the area covered.

Platelet adhesion and thrombus formation in response to collagen activation was evaluated during physiologic flow conditions in h2‐calponin KO platelets versus wild‐type control platelets that contain normal endogenous h2‐calponin. Four age‐, sex‐, and body weight‐matched mice pairs (h2‐calponin KO vs. wild‐type) were evaluated, and characteristics of the mice and confirmation of h2‐calponin expression status are shown in Table 1 and Fig. 3A respectively.

**Table 1.** Individual characteristics of mice sampled for whole blood thrombosis.

Mouse ID	Gender	Age (day)	Body weight (g)
KO1	Female	210	21.3
WT1	Female	204	22.2
KO2	Female	164	21.9
WT2	Female	165	26.3
KO3	Male	87	23.9
WT3	Male	87	26.2
KO4	Male	97	22.6
WT4	Male	97	23.7

Four pairs of mice were matched based on age, sex, and weight. WT, wild type; KO, h2‐calponin knockout.

Physiologic shear and pulsatility significantly impact platelet activation (Neeves et al. 2008; Nesbitt et al. 2009; White et al. 2014). A microfluidic‐based whole blood thrombosis assay was employed to simulate physiologic arterial flow conditions (shear stress of 15 dynes/cm^2^, pulse frequency of 1.67 Hz) to determine if h2‐calponin contributed to platelet activity in a clinically significant manner (See Materials and Methods section for detail). Whole blood preparations were fluorescently labeled, flowed through the microfluidic network over immobilized collagen, and monitored by serial fluorescent photomicroscopy to document platelet and thrombus accumulation during physiologic flow conditions (“All Thrombi” in Fig. 3B). Lag time and slope were obtained based on the kinetics of platelet / thrombus accumulation as described (see Materials and Methods section) and are shown in Fig. 3C and D. A significantly longer lag time was observed for combined platelet adhesion and thrombus accumulation in h2‐calponin KO group compared to WT controls (130.02 ± 3.74 sec and 72.95 ± 16.23 sec, respectively, *P* < 0.05), which reflects “all thrombi” or all individual platelet and thrombus interactions observed within the specified region of the viewing area (Fig. 3B,C, and D). The kinetics of individual thrombi accumulation was also analyzed by identifying 33 individual thrombi from WT and 33 individual thrombi from h2‐calponin KO mice (Fig. 3B, E, and F). These thrombi were all within the center of the viewing path and within the central third of the channel width where the shear environment of thrombus formation more consistent across different microfluidic channels (Fig. 3B). Despite the prolonged delay time observed in whole blood from h2‐calponin KO mice, the rate of thrombus accumulation was not significantly different compared to whole blood from wild‐type‐matched control mice (Fig. 3B, E, and F).

### H2‐calponin knockout mice have longer bleeding time and greater blood loss compared to controls

**Figure 4.**
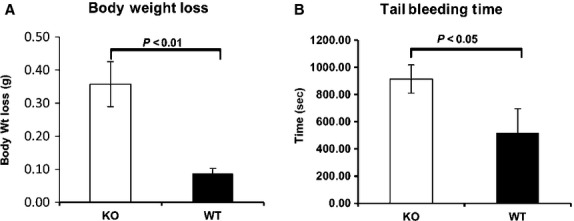
Mouse tail bleeding assay. Tail bleeding test was done with three pairs of gender‐ and age‐matched wild‐type and h2‐calponin knockout mice. Mice were anesthetized with sodium pentobarbital at 60 mg/kg body weight. Distal 10‐mm segment of tail was amputated with a surgical blade and the tail was immersed in a Falcon tube containing 50‐mL of isotonic saline prewarmed to 37°C. Bleeding time was recorded using a stop clock for 20 min. Body weight was measured right before and after the bleeding test. A significant body weight decrease (KO: 0.46 ± 0.08 g, WT: 0.08 ± 0.03 g, *P* < 0.01, (A) H2‐calponin knockout mice were observed compared to wide‐type mice. Tail bleeding time of calponin knockout mice was also significantly longer than that of wild‐type mice (KO: 1005.33 ± 35.18 sec, WT: 437.67 ± 219.82s, *P* < 0.05, (B).

A tail bleeding assay was performed to determine if there were any gross differences in hemostasis phenotype in h2‐calponin KO versus WT control mice (Fig. 4). We observed a statistically significant increase in both blood loss based on change in body weight (KO: 0.46 ± 0.08 g, WT: 0.08 ± 0.03 g, *P* < 0.01, Fig. 4A) and bleeding time (KO: 1005.33 ± 35.18 sec, WT: 437.67 ± 219.82 sec, *P* < 0.05, Fig. 4B) in h2‐calponin KO versus WT mice. These data demonstrate that time to hemostasis is prolonged in the h2‐calponin KO mice.

## Discussion

This is the first report of the h2 isoform of calponin presence and function in platelets. Although calponin has been identified in bovine and human platelets (Takeuchi et al. 1991; Meyer et al. 1996), the specific isoform had not been described prior to this report. Additionally, previous reports did not evaluate the functional role of calponin in platelet adhesion and thrombosis. H2‐calponin is known to associate with actin filaments and regulate actin filament activity in epithelial cells (Hossain et al. 2005, 2006), endothelial cells (Tang et al. 2006), fibroblasts (Hossain et al. 2005), and myeloid phagocytes (Huang et al. 2008). These prior studies of h2‐calponin may help to contextualize its potential role in platelets.

Based on previous observations of calponin in platelets and h2‐calponin in monocytes, which like platelets and erythrocytes are of the myeloid lineage, we initially hypothesized that the h2‐calponin isoform would be identified in both erythrocytes and platelets. However, h2‐calponin was not observed in erythrocytes by Western blotting or immunofluorescence. Erythrocytes form through the sequential steps of erythropoiesis, where the erythroblast progresses to a normoblast, which undergoes extrusion of the nucleus, hemoglobin accumulation, and release from the bone marrow into the peripheral circulation as a reticulocyte, and eventually becomes a mature erythrocyte (Harrison 1976). It is plausible that the h2‐calponin isoform may become degraded during the maturation process, resulting in the absence in mature erythrocytes observed in this report. Alternatively, the release of erythrocyte precursors from the adhesive bone marrow matrix into the peripheral circulation may result in downregulation of h2‐calponin production in much the same way circulating monocytes contain less h2‐calponin than matrix‐bound tissue macrophages. The expression of h2‐calponin is dependent on the tension built in the actin cytoskeleton against the stiffness of the immobilized matrix (Hossain et al. 2005), thus the removal of this signal upon transition of erythrocyte precursor from the bone marrow to the peripheral circulation may downregulate h2‐calponin transcription and/or translation. Platelets are fragments from parent megakaryocytes, thus more temporally associated with their nucleated progenitor, which may result in better retention of the h2‐calponin isoform in platelets compared to erythrocytes.

H2‐calponin appears to have diffuse cytoplasmic distribution throughout the platelet during quiescent conditions (Fig. 2A). This distribution is consistent with previous observations made with unspecified calponin isoforms in resting bovine platelets (Takeuchi et al. 1991) and human platelets (Meyer et al. 1996). Calponin has also been shown to colocalize with actin following platelet activation in suspension, as demonstrated by calponin and actin translocation to the submembrane following platelet activation (Takeuchi et al. 1991; Meyer et al. 1996). Since these platelets were spherical and not spread on immobilized collagen, h2‐calponin localization within lamellipodia and filopodia could not be documented. In the present study, h2‐calponin has a focal distribution throughout the leading edge lamellipodia when both human and mouse platelets are activated on immobilized collagen (Fig. 2C and E). This may represent focal areas of membrane attachment to the collagen substrate. F‐actin appears to be distributed more diffusely throughout the leading membrane edge, interrupted by the focal areas of calponin accumulation. Actin is known to facilitate attachment of glycoprotein VI and undergoes dynamic assembly and disassembly to facilitate membrane spreading. As in other nonmuscle cell types, h2‐calponin likely plays an important role in regulating this dynamic process of actin assembly and disassembly. Platelets are unique in that h2‐calponin regulation is exclusively posttranslational. Although previous studies show that calponin does not become phosphorylated following platelet activation (Meyer et al. 1996), it is possible that phosphorylation may occur during focal attachments required for platelet spreading on immobilized collagen. Further studies to elucidate the specific posttranslational regulatory mechanisms of h2‐calponin in platelets may reveal novel therapeutic targets to modify the interaction between h2‐calponin and actin, and ultimately platelet function.

Because the platelet cytoskeleton is integrally involved in regulating platelet function during *in vivo* shear conditions, dynamic platelet adhesion/thrombosis was evaluated in an in vitro microfluidic flow‐based thrombosis assay that simulates *in vivo* physiologic flow conditions. The lag time in whole blood from h2‐calponin KO mice was an average of 57.1 sec longer compared to whole blood from WT control mice. However, the rate of platelet accumulation during the rapid phase (slope of the linear segment of the curve) was similar in both groups (Fig. 3). These are novel findings, as h2‐calponin has not been previously described to affect dynamic platelet adhesion and thrombosis during physiologic flow conditions. We also observed statistically significant increases in bleeding time and blood loss in h2‐calponin KO mice compared to WT control mice, demonstrating phenotypic differences in hemostasis (Fig. 4). The tail bleeding assay measures time to hemostasis in a mixed arterial and venous bleeding model, and the absence of h2‐calponin increases time to achieve hemostasis in this environment. Thrombosis will also need to be evaluated in the isolated arterial circulation, such as the FeCl_3_ arterial thrombosis model (Vaezzadeh et al. 2014), to better understand the role of h2‐calponin during in vivo arterial thrombosis. We are actively exploring this direction.

Macrophages demonstrate faster migration and increased phagocytosis in the absence of h2‐calponin (Huang et al. 2008), both processes require highly dynamic actin cytoskeletal regulation. It is possible that platelets are less able to form early attachments to collagen during physiologic flow conditions, but are able to increase adhesion at a normal rate once a critical mass of adherent platelets are available to facilitate capture of flowing platelets. These data suggest that the absence of calponin may slow the initiation of platelet adhesion and aggregate formation without affecting the rapid phase of platelet accumulation on collagen. H2‐calponin as a therapeutic target that can reduce pathologic thrombus initiation while minimizing spontaneous bleeding complications could have significant clinical utility, such as in postoperative congenital heart surgery patients or any preoperative surgical candidate with an indication for platelet inhibition. As a result, further studies are required to better understand how h2‐calponin regulates platelet function during physiologic flow conditions.

## Acknowledgments

We thank M. Moazzem Hossain in the Department of Physiology at Wayne State University School of Medicine for breeding and maintenance of the h2‐calponin KO mice, and M. Zidan in the Department o Pediatrics at Wayne State University School of Medicine for assistance with statistical analysis.

## Conflict of Interest

None declared.
